# Parasite (*Anisakis* spp.) Load and Its Relationship with Diet in Common Dolphins (*Delphinus delphis*) Along the Coast of Galicia (NW Spain)

**DOI:** 10.3390/ani16040682

**Published:** 2026-02-21

**Authors:** Elisa Rueda-Díez, Gema Hernandez-Milian, Alberto Hernandez-Gonzalez, Silvina Ivaylova Tsanicheva, Sébastien T. Jacquot, Marie A. C. Petitguyot, Paula Gutiérrez-Muñoz, Pablo Covelo, Xabier Pin, Alfredo López, Graham J. Pierce

**Affiliations:** 1 Facultad de Ciencias del Mar, Universidad de Vigo, Campus Do Mar, 36210 Vigo, Spain; 2 Instituto de Investigaciones Marinas (IIM-CSIC), 36208 Vigo, Spain; 3 Centro Oceanográfico de Vigo, Instituto Español de Oceanografía (IEO-CSIC), 36390 Vigo, Spain; gema.hernandez@ieo.csic.es (G.H.-M.);; 4 Faculté des Sciences et Technologies, La Rochelle Université, 17000 La Rochelle, France; 5 Coordinadora para o Estudio dos Mamíferos Mariños (CEMMA), 36380 Gondomar, Spain; 6 Centro de Estudos do Ambiente e do Mar (CESAM), Universidade de Aveiro, 3810-193 Aveiro, Portugal

**Keywords:** *Delphinus delphis*, common dolphin, Galician coast, *Anisakis* spp., parasite load, diet

## Abstract

The common dolphin (*Delphinus delphis*) is one of the most abundant small cetaceans along the Galician coast and a definitive host for the nematode parasite *Anisakis*, which is transmitted to cetaceans through the food chain. This study aimed to analyse which factors, including dolphin diet, affect the parasitic load. Samples of stomach contents from stranded dolphins along the Galician coast (2004–2024) were examined. The number of parasites was counted, and the contribution of different prey species to the diet was analysed based on hard remains. Generalised Additive Models (GAMs) were used to assess the relationships between parasitic load (number of *Anisakis* in the stomach) and various putative explanatory variables (e.g., year, month, size, sex, latitude, body condition, cause of death and diet of the dolphins). Results showed an increase in parasitic load over the years and a seasonal pattern, with numbers peaking in the first months of the year. A significant positive relationship was found between dolphin length and *Anisakis* load. In addition, dolphins that died from bycatch had the lowest parasitic loads. The numbers of Atlantic mackerel (*Scomber scombrus*) and blue whiting (*Micromesistius poutassou*) in the stomach had a significant effect on parasite load: parasite abundance decreased as the numbers of these prey species in the stomach increased. This result confirms the influence of diet on *Anisakis* load, although it does not reveal which species contribute the most to the parasite load. The study offers insights into how diet and other ecological factors influence the parasitic load in *D. delphis*.

## Simple Summary

The common dolphin (*Delphinus delphis*) is one of the main cetacean species along the Galician coast and acts as the final host for the nematode parasite *Anisakis*. These parasites are acquired through the consumption of infected fish and cephalopods. Understanding the relationship between these parasites and *D. delphis* can help us assess the status of *Anisakis* populations in the marine ecosystem and, since this parasite can be transmitted to humans, contribute to improving public health safety by providing insights into potential risks. This study aimed to investigate which factors, including diet, influence the number of parasites in the stomachs of *D. delphis*. Results revealed that the number of parasites has increased over the years and is higher in the first months of the year. Larger dolphins had more parasites, while dolphins that died due to incidental capture in fishing gear had fewer parasites. Dolphins with a higher number of Atlantic mackerel and blue whiting in their stomachs showed a lower parasitic load. This confirms that the diet might be an important factor in determining *Anisakis* load in *D. delphis* even if it does not clearly identify which prey species contribute most to the *Anisakis* load in dolphins. This study offers insights into how diet and other ecological factors influence the parasitic load in *D. delphis*.

## 1. Introduction

Cetacean species play a crucial role in marine ecology and act as final hosts for *Anisakis* nematodes Dujardin, 1845 (hereinafter “*Anisakis*”), a globally distributed zoonotic nematode transmitted through the food chain. The parasite’s life cycle also involves planktonic crustaceans as primary hosts and fish and cephalopods as paratenic hosts. When cetaceans ingest infected prey, some of the *Anisakis* larvae (in the L3 stage) attach to the stomach walls, develop into L4 larvae and adult stages, reproduce, and are eventually excreted in the faeces [1,2,3]. Although cetaceans do not usually die directly from *Anisakis* infection [4], this parasite can cause various pathologies, such as gastric and intestinal haemorrhages; ulcerative, fibrous, and granulomatous gastritis; oesophagitis; or obstructions [4,5]. A high parasitic load of *Anisakis* in the stomach can also lead to mild anaemia and limit food intake, increasing the risk of starvation [6].

Infection of fish and cephalopods by *Anisakis* not only affects dolphins but is also a public health concern. Humans can become infected through the consumption of raw or undercooked fish [2], thus becoming accidental hosts in which the parasite can live for approximately 3 weeks, although it cannot reproduce [2,3]. This condition, known as anisakiasis, can cause severe gastrointestinal symptoms such as abdominal pain, vomiting, and diarrhoea. Severe allergic reactions, which can lead to anaphylaxis in sensitised individuals, can also occur not only after eating fish containing live parasites but also after ingesting dead *Anisakis* or their proteins [7,8]. Of the nine species within this genus, only two (*A. simplex,* Rudolphi, 1809, and *A. pegreffii,* Campana-Rouget & Biocca, 1955) have been confirmed as zoonotic pathogens [2], and both are present in Spanish waters [3].

The common dolphin (*Delphinus delphis;* Linnaeus, 1758) is one of the most abundant cetacean species in the Atlantic Ocean. Based on results from the SCANS IV survey in 2022, its abundance in the northeast Atlantic is estimated to be 439,212 individuals (309,153–623,987). It is also one of the most commonly observed species in the coastal and oceanic waters of Galicia (NW Spain) [9]. Every year, hundreds of *D. delphis*, mostly dead animals, are stranded in Galician coast; these strandings exhibit an irregular distribution throughout the year, with the highest number of strandings normally occurring in the first quarter of the year [10]. Strandings in this area are likely influenced by several factors such as winds [10,11,12], species-specific distribution patterns and/or differences in mortality, e.g., in fishery bycatch rates [13], and accessibility of the coastline [10]. The probability of detecting a stranded dolphin depends on multiple factors, including the location of death, carcass buoyancy, drift dynamics, and observation effort [12], as well as the resources available to the stranding network to visit, examine and necropsy carcases at different locations and at different times of year. All these factors may result in a sample that is not wholly representative of the animals that die each year [14]. It should also be evident that the dead animals are also not representative of the living population, neither in terms of health status (there will inevitably be a higher proportion of animals that were in poor health among the dead animals) nor age structure (given that mortality rate in cetaceans is expected to be age-related) [15]. Note, however, that the age structure of the living population can be inferred from the age distribution of dead animals by creating a life table (e.g., [15]).

*Delphinus delphis* feed on a wide variety of prey, including epipelagic and mesopelagic fish and cephalopods [16,17,18]. Among their most important prey are energy-rich species such as sardine (*Sardina pilchardus,* Walbaum, 1792), horse mackerel (*Trachurus* spp., Rafinesque, 1810), and mackerel (*Scomber* spp., Linnaeus, 1758), as well as less energy-rich species like European hake (*Merluccius merluccius,* Linnaeus, 1758), blue whiting (*Micromesistius poutassou*, Risso, 1827), and pouting (*Trisopterus* spp., Rafinesque, 1814). The most common cephalopod families in their diet are Loliginidae (Lesueur, 1821), Ommastrephidae (Steenstrup, 1857), and Sepiolidae (Leach, 1817) [17,19,20]. The presence of *Anisakis* larvae has been reported in many of these species in the northwestern Iberian Peninsula (Appendix A, Table A1). *Merluccius merluccius*, *T. trachurus*, *M. poutassou* and *S. scombrus* show a high prevalence of *Anisakis* [21,22,23,24,25,26,27,28,29,30,31,32,33], while the intensity of infection tends to be highest in *M. merluccius* [21,24,26,27,28,29,30].

Since *Anisakis* is ingested via food, we would expect diet, for example as indicated by stomach contents, to have an impact on the number of *Anisakis* present in the stomach. Similarly, because the amount of *Anisakis* ingested will depend on the amount of food eaten, it is likely that larger dolphins will ingest more *Anisakis*. It is generally considered that, among stranded animals, trauma deaths would be expected to be more representative of the health status of living animals if compared with animals that died due to causes such as disease. In Galicia, fishery bycatch is by far the most frequent cause of trauma deaths in cetaceans. Hence, comparing bycaught versus non-bycaught animals provides a simple way to investigate the relationship between health status and parasite burden. This is not to say that bycaught animals are always healthy, only that on average their health status will be better than that of animals dying from other causes. Another source of information on condition, and hence related to health, that is frequently available from stranded animals is blubber thickness.

The aims of this study were to: (i) determine the number and proportion of adult worms in *D. delphis* stomach contents, (ii) analyse the factors (biological and environmental) affecting the abundance and average size of *Anisakis* in the stomachs of *D. delphis*; and (iii) examine the relationship between diet composition and *Anisakis* load in this cetacean species, evaluating whether higher consumption of prey species with higher parasite loads results in a higher parasitic burden in *D. delphis*. This approach may provide new insights into the life cycle and population dynamics of the parasite *Anisakis*. In addition, it may reveal infection patterns related to the consumption of intermediate host species, which could be useful for assessing potential risks to human health.

## 2. Materials and Methods

Individuals of *D. delphis* found dead along the Galician coast between 2004 and 2024 were necropsied by the NGO Coordinadora para o Estudo dos Mamíferos Mariños (CEMMA) following the European Cetacean Society’s standard protocol [34]. The information collected on each animal normally included stranding date, location, body length, sex and blubber thickness (body condition); cause of death (COD) was diagnosed when possible, distinguishing between animals with clear signs of bycatch and those with no evidence of bycatch. Samples collected also included stomach contents.

The data and samples analysed here were obtained from a total of 117 stranded *D. delphis*. Parasitological information was available for all individuals while dietary information was available for 90 dolphins, since 27 individuals did not have food remains in the stomach.

**Figure 1:**
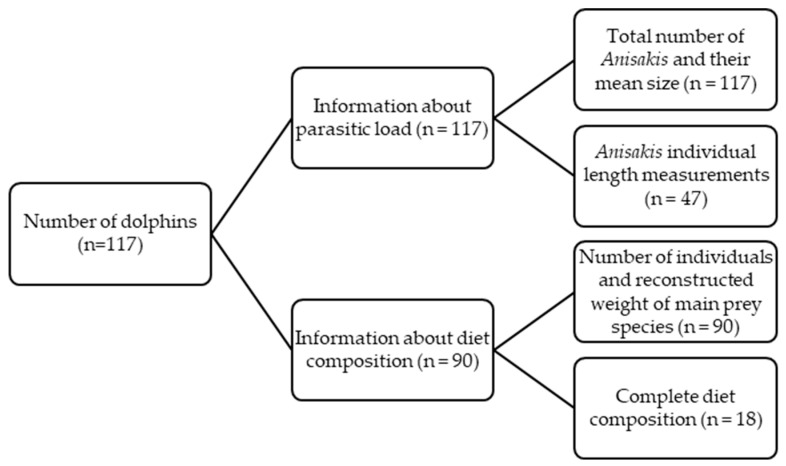
Summary of sample sizes used for parasitological and dietary analyses in this study.

Parasitological data were collected in two stages. Data for 70 dolphins were collected by S.T.J. in 2021 and data for a further 47 dolphins were collected by E.R.-D. in 2023–2024, in this case following a revised sampling procedure to improve the estimation of the size distribution (see Section 2.1). Dietary data for 72 of the dolphins were collected by A.H.-G. [18] and E.R.-D. collected dietary data from a further 18 dolphins. These 18 animals represent the most recently available dietary information for this species in this area and are analysed in detail here (see Section 2.2) (Figure 1).

### 2.1. Sampled Dolphins

**Figure 2:**
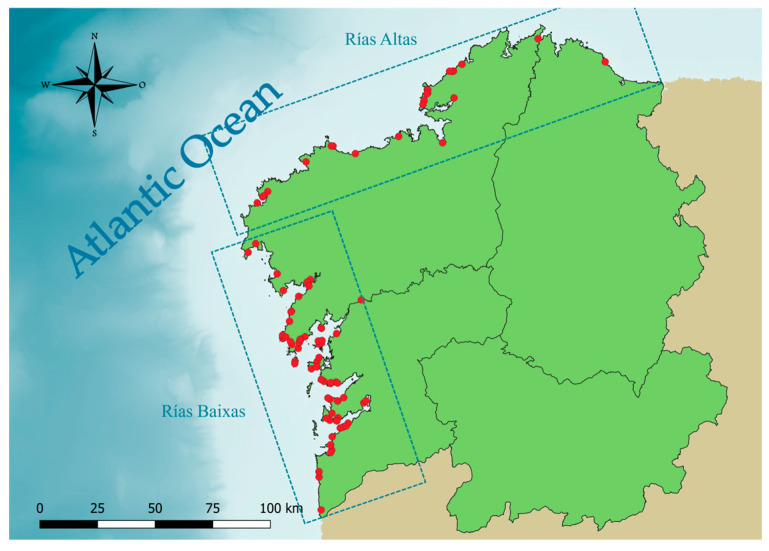
Map of the study area. Red dots are the stranding locations of the sampled *D. delphis* (n = 117).

Of the 117 *D. delphis* collected between 2004 and 2024 (Table 1), 53 dolphins (45%) were females and 64 (55%) were males; 66 out of 117 (56%) had stranded in the first quarter of the year. In relation to COD, 46 individuals (39%) showed signs of bycatch, 55 (47%) showed no signs of bycatch, and no information on COD was available for the remaining 16 dolphins (14%). Body length ranged from 134 to 237 cm, with an average length of 187.7 cm for males and 188.8 cm for females. Approximately three-quarters of the dolphins (87, 74%) were collected in the Rías Baixas area, while the remaining 30 individuals were found in the Rías Altas (Figure 2).

### 2.2. Anisakis Data

For the first 70 dolphins, analysed by S.T.J., all *Anisakis* were counted and measured when the parasite load was low. For highly infested samples, a subsample (of between 30 and 100 worms) was measured for length, weight, and volume. The remaining parasites were weighed and their total volume recorded, allowing estimation of the total number present. In the remaining 47 samples, from 2008 to 2024, all *Anisakis* individuals were counted. To estimate the number of adults and larvae present in the stomach, we calculated the size distribution of the worms. We used a stratified sampling protocol to ensure that the less common larger worms were adequately sampled: the worms were first categorised by visual examination as large or small based on whether they appeared to be more or less than 3.5 cm in length, since adults are typically greater than 3.5 cm in length [35]. The number of worms in each category was then counted. From each size class, 100 individuals were subsequently measured to create the overall size distribution. From this overall size distribution, we derived both the number of adults and the average size of worms in the stomach.

### 2.3. Diet Data

The diet of 18 *D. delphis*, stranded between 2019 and 2024, was described through the analysis of their stomach contents. Previous dietary information (abundance and reconstructed weight of main prey species) for a further 72 dolphins, collected by A.H.-G. using similar methods, was included in the statistical analyses.

Fish species were identified from sagittal otoliths, jaw bones (dentaries, premaxillae, and maxillae), and vertebrae, using identification guides (for otoliths: [36,37]; for bones: ref. [38] and photographs from a reference collection of fish otoliths and skeletons (Hernandez-Milian and Pierce, unpublished)). Since most of these structures (except for vertebrae) are paired, in samples with few hard remains, otoliths and/or jaw bones from both sides of the head were matched to estimate the number of fish more accurately. In samples with abundant remains, structures from both sides were counted, and the higher number was recorded. When different types of hard remains were found for the same species, the final number of individuals was estimated as the highest value based on the available information.

Fish size was estimated using measurements from otoliths and specific bones (dentaries, premaxillae, and caudal vertebrae) (Appendix B, Table A2), applying published regression equations (e.g., [36,39,40]) (Appendix B, Table A2). Measurements were taken with a binocular microscope and a millimetre ruler for otoliths and small bones. Callipers were used for bones larger than 1 cm in length.

Cephalopod species were identified from the beaks (mainly the lower beaks) using identification guides [41,42,43] and reference material. Their presence was also determined from their eyes. Abundance was estimated by counting all beaks, attempting to match lower and upper beaks from the same individuals or using the higher count when pairing was not possible. Cephalopod size was estimated from beak measurements (Appendix B, Table A2) using published regression equations (Appendix B, Table A2), with lower beak measurements being the primary focus for size estimation.

Four standard indices were used to assess the relative overall importance of each prey type in the diet:

1. Percentage Frequency of Occurrence (F%):
  (1) $$F\%=\left(\frac{F_{i}}{F_{t}}\right)\times 100$$
  where F_i_ is the number of stomachs containing prey type i and F_T_ is the total number of stomachs analysed.
2. Prey Number Percentage (N%):
  (2) $$N\%=\left(\frac{N_{i}}{N_{T}}\right)\times 100$$
  where N_i_ is the number of individuals of prey i and N_T_ is the total number of all prey individuals found in all stomachs.
3. Prey Weight (reconstructed weight) Percentage (W%):
  (3) $$W\%=\left(\frac{W_{i}}{W_{T}}\right)\times 100$$
  where $W_{i}$ is the total reconstructed weight of prey i in all stomachs and $W_{T}$ is the total reconstructed weight of all prey species in all stomachs.
4. Percentage Index of Relative Importance (%IRI):
  (4) $$IRI\%=\left(\frac{{IRI}_{i}}{{IRI}_{T}}\right)\times 100$$
  where ${IRI}_{i}$ is the Index of Relative Importance of prey taxon i [44], calculated as
  (5) $$IRI=\left(N\%+W\%\right)\times F\%$$
  and ${IRI}_{T}$ is the total sum of all ${IRI}_{i}$ numbers.

This index combines the previous three indices and represents the overall relative importance of each prey type in the diet.

### 2.4. Statistical Analysis

Data analysis was conducted using the software R (4.3.2) [45]. Generalised Additive Models (GAMs) were applied to quantify the influence of several explanatory variables on the *Anisakis* load. The response variable used in the GAMs was the total number of *Anisakis* found in each stomach, assumed to follow a negative binomial distribution. For those continuous explanatory variables not expected to have a complex relationship with *Anisakis* load, the complexity of the fitted smoother was constrained by setting k = 4. Month was treated as a cyclic variable by setting bs = “cc”. Given the relatively small dataset, interactions between explanatory variables were not investigated. In all cases, a backward selection approach was applied, and the final (optimal) model was chosen based on the Akaike Information Criterion (AIC) [46].

For the first set of models (base model), the explanatory variables included were: year, month, dolphin length, sex and latitude. These explanatory variables were selected due to their possible influence on parasite load and because data were available for all samples.

Because data were not available for all samples on blubber thickness (commonly used as an indicator of health condition) and COD (as bycatch versus non-bycatch, i.e., a binary variable of yes/no), a second set of models with a smaller sample size was fitted. In these models, both blubber thickness and COD were included as explanatory variables, in addition to those already considered in the first set of models.

Finally, to examine the relationship between diet and parasitic load, a third set of models was fitted, in which the total numbers of each of the main prey species found in the dolphin stomachs were added to the base model as additional explanatory variables. The prey species selected for the models were *M. merluccius* (HKE), *M. poutassou* (WHB), genus *Trisopterus* (TRX), family Clupeidae (CLP), *S. scombrus* (MAC), and *T. trachurus* (HOM). As with data on blubber thickness and COD, dietary information was not available for all dolphins, leading to reduced sample sizes in these models.

## 3. Results

### 3.1. Anisakis Load and Average Size

An estimation of the numbers of “adult” (>3.5 cm length) and “larvae” (≤3.5 cm) *Anisakis* present in the subsample of 47 dolphins showed that most of the *Anisakis* present in the stomachs (81.5%) were probably larvae (<3.5 cm), while 18.5% were probably adults (Table 2).

**Figure 3:**
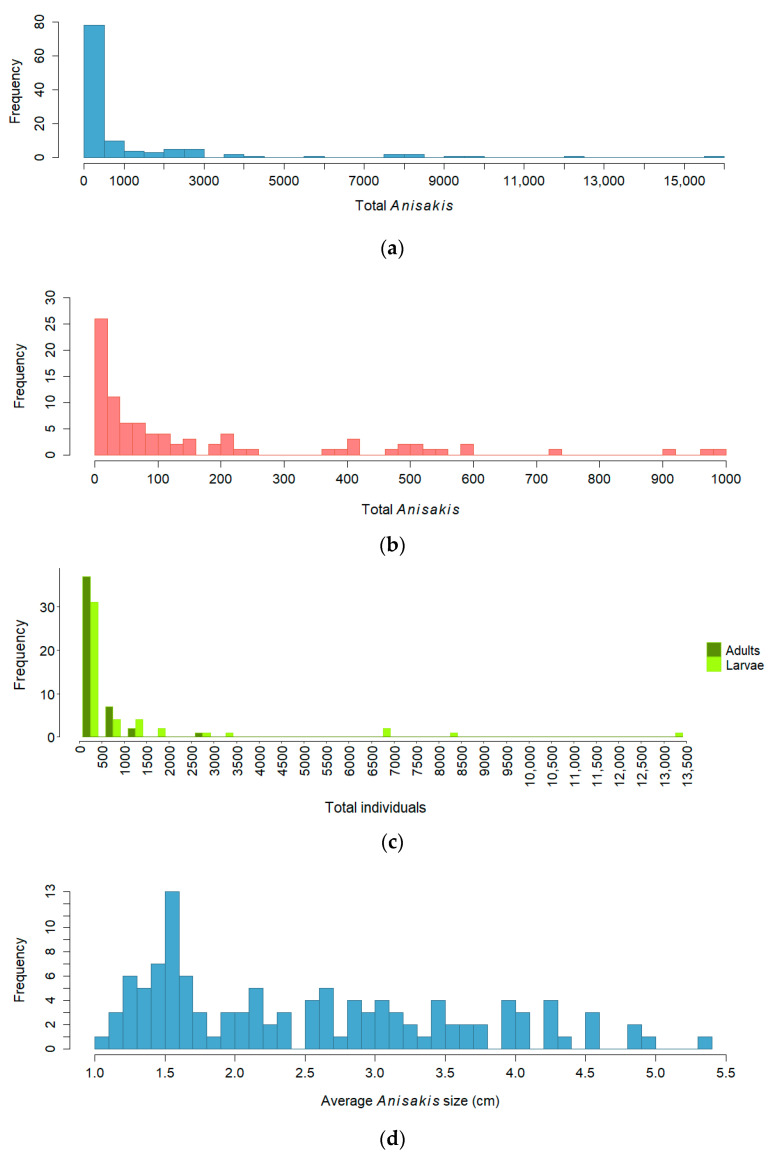
Frequency distributions of the total number of *Anisakis* and their average size in each stomach. (**a**) All samples analysed, (**b**) samples with fewer than 1000 parasites, (**c**) adults and larvae, (**d**) average size of *Anisakis* from all samples. These frequency distributions refer to all 117 stomachs except for c, which refers to the 47 stomachs for which stratified subsampling was carried out.

The distribution of the estimated total number of *Anisakis* present in individual stomachs is highly right-skewed. It should be noted that one animal had around 16,000 worms, few animals had more than 1000 and the majority had fewer than 100 worms in their stomachs (Figure 3a,b). A similar right-skewed pattern was observed when adults and larvae were analysed separately (Figure 3c). However, it can be observed that adults were present in lower abundances in dolphin stomachs. Figure 3d shows the average size of *Anisakis* per stomach for all 117 samples. While the average parasite size varied considerably, small *Anisakis* were most numerous in almost all dolphins.

### 3.2. Diet Composition

In the 18 recent *D. delphis* stomachs (from 2019 to 2024) analysed in this study, in total at least 878 individual prey species were identified based on 1528 prey hard parts. The reconstructed diet composition is summarised in Table 3.

The diet of these 18 dolphins consisted mainly of fish (F% = 88.9; N% = 87.8; W% = 97.6 and IRI% = 96.2). Among 21 fish species belonging to 15 families, the species with the highest frequency of occurrence were *M. poutassou* (F% = 33.3), *M. merluccius* (F% = 27.8), *T. trachurus* (F% = 22.2) and *Trisopterus* spp. (F% = 22.2). The most numerically important fish prey were *M. poutassou* (N% = 27.11), Gobiidae (N% = 17.3), *Argentina* spp. (N% = 17), and *Trisopterus* spp. (N% = 12.2). In terms of importance by reconstructed weight, the most important prey by far was *M. poutassou* (W% = 91.9), followed by *M. merluccius* (W% = 2.36). Finally, the IRI% indicated that the most important prey was *M. poutassou* (IRI% = 74.8) (Table 3).

Cephalopods were also present in the stomachs but generally less important than fish (F% = 44.4; N% = 12; W% = 2.4 and IRI% = 3.7). Loliginidae was the most important cephalopod family in the diet (F% = 33.3, N% = 6.6, W% = 1.4, and IRI% = 5.0), while *Loligo* was the most important genus (F% = 33.3, N% = 5.3, W% = 1.4, and IRI% = 4.2) (Table 3 and Appendix C, Table A3b).

The estimated lengths of the fish eaten ranged from 2 to 319 mm with *M. merluccius* and *M. poutassou* being the largest species. The estimated reconstructed weights ranged from 0.0004 to 1571.5 g, with the maximum reconstructed weight being recorded for *M. poutassou*. In the case of cephalopods, the reconstructed sizes ranged from 4 to 278 mm, and the reconstructed weight ranged from 0.3 to 469.6 g, with the largest and heaviest cephalopod prey belonging to the genus *Loligo* (Appendix C, Table A3b). It is important to note that the minimum reconstructed lengths and weights obtained are likely to be underestimates (see Section 4).

Combining the new and previously collected dietary data, and thus bringing the sample size up to 90, indicated that, in general, the same prey species were identified as important in the larger dataset (Table 4): *M. poutassou* was the most important species in terms of number, reconstructed weight and relative importance (N% = 73.7; W% = 75.6; %IRI = 79.5). In terms of frequency of occurrence, *M. merluccius* (F% = 44.4) and *M. poutassou* (F% = 43.3) were almost equally important.

### 3.3. Factors Affecting Anisakis Load

#### 3.3.1. Effects of Various Variables on the Total *Anisakis*

**Figure 4:**
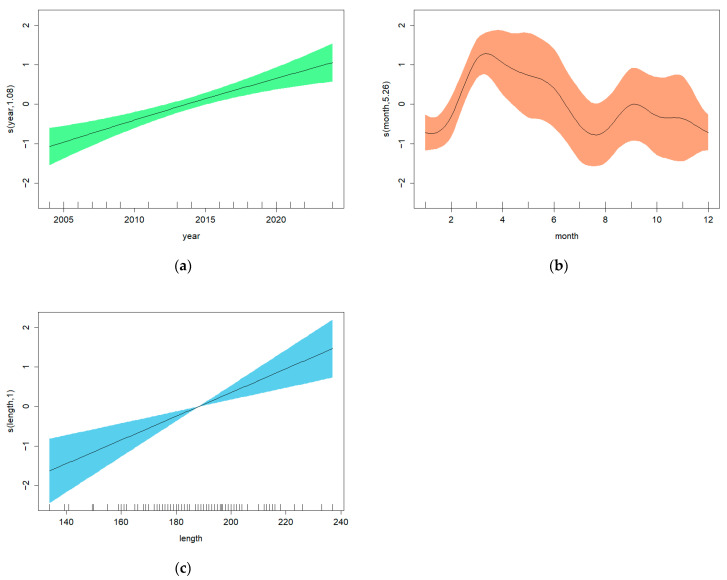
Optimal GAM 1: plots showing the effects of explanatory variables on the total number of *Anisakis* by (**a**) year, (**b**) month and (**c**) length. Fitted effects (solid lines) with 95% confidence intervals (shaded areas).

**Figure 5:**
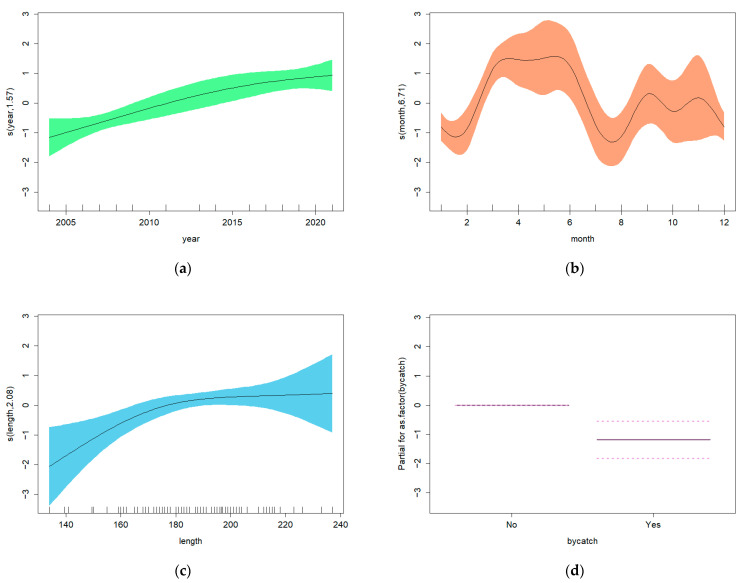
Optimal GAM 2: plots showing the effects of explanatory variables on the total number of *Anisakis* by (**a**) year, (**b**) month, (**c**) length, and (**d**) COD. Fitted effects (solid lines) with 95% confidence intervals (shaded areas or dashed lines).

The optimal GAM based on the baseline explanatory variables showed significant relationships between the total number of *Anisakis* and the variables year, month and dolphin length (Table 5, GAM 1; Figure 4). For the second set of GAMs, run on the subset of 101 *D. delphis* for which the variables blubber thickness and COD were available, there was no significant effect of blubber thickness, but COD had a significant effect on the total number of *Anisakis* (Table 5, GAM 2; Figure 5).

The graphical representations of GAM 1 (Figure 4a) show a general increase in the total number of *Anisakis* from 2004 to 2024. However, in GAM 2, the upward trend ceases to be significant from 2015 to 2021 (a horizontal line would fit within the 95% confidence limits) (Figure 5a). In both GAM 1 and GAM 2, there is an increase in parasite number during the first months of the year (January–May), followed by a decline until August. The apparent increase from August to September is probably non-significant (Figure 4b and Figure 5b). In GAM 1, dolphin length showed a significant positive relationship with the total number of *Anisakis*, with larger animals having more parasites in their stomachs (Figure 4c). In GAM 2, an upward trend is seen, but it becomes non-significant above a body length of around 180 cm (Figure 5c). The results of GAM 2 also showed a lower number of parasites in bycaught animals (Figure 5d).

#### 3.3.2. Relationship Between *Anisakis* Load and Diet

**Figure 6:**
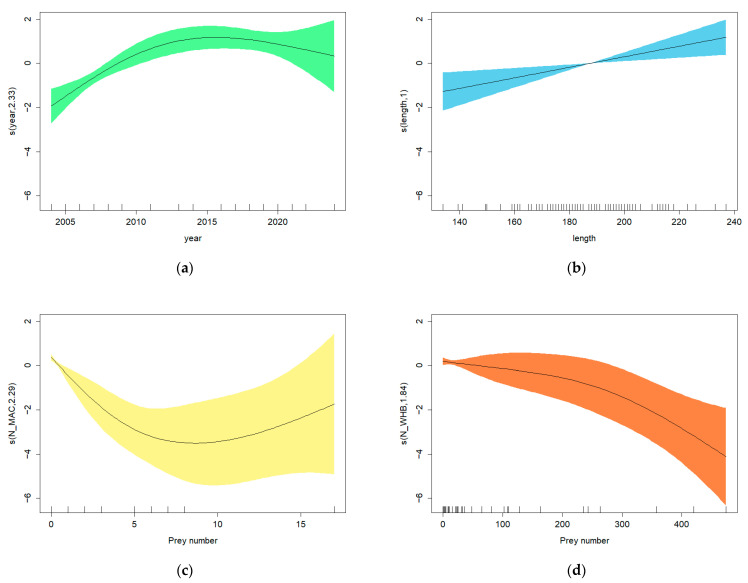
Optimal GAM 3: plots showing the effects of explanatory variables on the total number of *Anisakis* by (**a**) year, (**b**) length, (**c**) number of *S. scombrus* and (**d**) number of *M. poutassou*. Fitted effects (solid lines) with 95% confidence intervals (shaded areas).

The optimal model including the dietary information (Table 5, GAM 3) showed a significant relationship between *Anisakis* load and the variables year, dolphin length, number of *S. scombrus* in the stomach and number of *M. poutassou* in the stomach. The graphical representation for this last model (Figure 6) showed an increase in the total number of *Anisakis* from 2004 until around 2015, followed by a non-significant decline until 2022 (Figure 6a). Dolphin length had a significant positive effect on *Anisakis* load (Figure 6b). The significant diet variables were the number of *S. scombrus* in the stomach (N_MAC) (Figure 6c) and the number of *M. poutassou* (N_WHB) (Figure 6d). A decrease in the total number of *Anisakis* can be observed as the number of *M. poutassou* in the stomach increases. In the case of the *S. scombrus*, the relationship between *Anisakis* load and number of prey in the stomach is also significant and negative at low prey abundance. However, at higher *S. scombrus* numbers, confidence limits are very wide and a clear trend is not visible.

## 4. Discussion

### 4.1. Anisakis Sizes

The estimated numbers of individuals with lengths above and below 3.5 cm, and the distribution of average sizes of *Anisakis*, showed that most of the parasites in the stomachs were small and were probably in larval stages according to the morphological characteristics described by Grabda [35]. This result can be understood in terms of the life cycle of *Anisakis* and the rates of ingestion, maturation and egestion. Through the consumption of infected prey, L3 larval stages of *Anisakis* enter the definitive host and some attach to the gastric mucosa, where they continue their development into L4 larvae and adults [1,2,3]. However, it is likely that many individuals fail to successfully complete development and are destroyed or expelled. In addition, adult worms are eventually eliminated after completing their life cycle, whereas new larvae continuously enter the host with prey, leading to a higher proportion of larval stages in the stomach. This can be observed in the distribution of adult and larval *Anisakis*, which shows that adults are predominantly found at low abundances, while larvae can reach higher numbers.

### 4.2. Diet Composition

Both in the present study and in previous studies [17,18], it was observed that fish species make up the bulk of the *D. delphis* diet, with *M. poutassou* standing out as the most important prey for *D. delphis* along the Galician coast, especially in terms of biomass ingested. *Sardina pilchardus*, which is the main prey of *D. delphis* in Portuguese waters [20], has decreased in the diet of dolphins in Galician waters since 1995 [17,18] and in the present study, it did not appear as an important prey species. The increase in the importance of *M. merluccius* [17,18] and the presence of gobies in the diet [18] were also observed in this study, although unlike previous records, the *Trisopterus* genus proved to be quite important. Cephalopods continue to appear as a minor part of the diet, and among them, the genus *Loligo* remains the most important [17,18].

It is important to note that some published regression equations are not very suitable for estimating the size of smaller individuals of some prey species, as they were calculated using larger individuals and the assumption of a linear relationship between fish length and otolith length is probably not justified when considering the whole size range. This led to results such as estimated lengths of 1.95 mm or weights of 0.0004 g, which are evidently not possible and will have introduced some bias regarding the reconstructed total weight of the consumed prey. In addition, the erosion of otoliths caused by digestion modifies their shape and, more importantly, reduces their size and may thus affect the calculation of fish size [47,48]. It would be useful to update the existing regression equations, increasing the size range of the fish and cephalopods used to construct them, making an effort to include the smallest individuals and taking into account the likely non-linearity of these relationships.

### 4.3. Factors Affecting Anisakis Load

The *Anisakis* load in *D. delphis* stomach is usually fewer than 100 worms; however, considering the number of *Anisakis* that might be ingested daily through their prey (Table 1), relatively few of them may be retained in the stomach for a long period of time. Further investigation should be undertaken on worms closer to maturation and/or those that manage to attach well to the stomach wall. It would also be interesting to further investigate which individual worms reach maturity and lay eggs and what factors determine their reproductive success. Very high parasite loads (several thousand *Anisakis*) were seen in a few individual dolphins, and it seems likely that such loads do imply accumulation, with reduced egestion, and might indicate poor health (although the question then arises as to whether parasite accumulation is a cause or consequence of poor health).

The total number of *Anisakis* parasites present in the dolphin stomachs appears to be influenced by several factors. All optimal models included the year as a significant factor, generally showing an increase in parasite abundance up until approximately 2015, although the trend since 2015 differs between models (Figure 4a, Figure 5a and Figure 6a). Several studies have already suggested that the *Anisakis* population may have increased in the area in recent years. Pons-Bordas et al. [49] observed an increase in gastric ulcers caused by this parasite in stranded dolphins between 2017 and 2018 on the Galician coast compared to those stranded between 1991 and 1996. Lino et al. [50] reported an increase in *Anisakis* numbers in dolphin stomachs up until 2020 in dolphins stranded along the Portuguese coast. These results also align with the rise in *Anisakis* abundance detected in fish and invertebrates between 1962 and 2015, according to a bibliographic review [51].

Given this information, the levelling off of or possible decline in the total number of parasites found in stomachs of stranded dolphins since 2015, as suggested by the models which accounted for the effects of COD and diet, is potentially interesting. This possible change in the trend could be related to changes in dolphin feeding habits due to fluctuations in prey populations [17]. Another factor that may have influenced the trend is changes in fishing practices, for example, the implementation of Regulation (EC) No 1069/2009, which prohibits the discarding at sea of offal showing signs of infection. It is possible that the gutting of fish on board and the discarding of the resulting offal (containing live *Anisakis* larvae) contribute to the spread of this parasite through the ecosystem, although it may help to reduce the amount of *Anisakis* in fish intended for human consumption [52]. Various ways of treating fish offal to prevent viable *Anisakis* from returning to the marine food chain have been proposed and tested (e.g., [53]). Therefore, the implementation of this regulation in 2011 could have led to a decrease in the parasite load in dolphins. However, further studies are needed both to verify the current trend in *Anisakis* load in *D. delphis* and to assess the factors that have influenced *Anisakis* abundance (in all of its host species) during this period.

Seasonal differences in *Anisakis* loads were also observed, and an increase in parasite abundance during the first months of the year (Figure 4b and Figure 5b) was found to be statistically significant. As seen in other studies, there is an increase in *Anisakis* abundance in spring and a decrease in winter [50,54]. Strømnes & Andersen [54] associated this increase in parasite numbers with the spring bloom; the rise in primary production triggers an effect throughout the food chain, leading to an increase in the abundance of *Anisakis* in intermediate hosts and, therefore, a higher probability of infection when feeding on them. A similar explanation could apply in Galician waters, as significant seasonal variations in primary production also occur in this region [55,56,57].

A further significant variable in explaining the total number of *Anisakis* was mortality due to bycatch. This refers to the COD of the dolphin—whether it was clearly due to bycatch or if there was no evidence of this. Given that bycaught dolphins tend to have been healthier animals than, for example, those that died due to poor health, and may thus have had high feeding rates, it is plausible that bycaught individuals would also present a higher abundance of parasites. However, the result obtained showed a significantly higher parasite load in dolphins with no evidence of bycatch (Figure 5d). It could be suggested that healthy dolphins are able to eliminate (egest) more *Anisakis.* Interpretation of the modelling results is necessarily speculative. There are still uncertainties regarding the effects of *Anisakis* infection in dolphins, indeed whether high parasite load can cause poor health, or vice versa, and the answer may differ between individual dolphins. In addition, caution is needed when interpreting analysis based on a relatively small sample size. Although dolphins generally do not die directly from *Anisakis* infection [4], it does cause multiple pathologies [4,5] and even anaemia or a higher risk of starvation [6]. It can thus reduce an animal’s physical fitness [58] and ultimately compromise its survival. In addition, animals in poorer health have weaker immune systems [59], which facilitates infection; this could explain why dolphins that die from infectious diseases, for example, have a higher parasite load. Another possibility is that healthy dolphins, with fewer parasites, are more likely to approach fishing nets to feed, which could lead to their accidental capture. Further study is needed on the relationship between the health status of cetaceans and their degree of infection, as well as the consequences of high parasite loads, to determine whether and how this interferes with their life.

As expected, there is a positive relationship between the size of the cetacean and the total number of parasites in its stomach. This is likely due to a higher daily food intake and/or the consumption of larger prey by larger cetaceans [20], which could lead to the ingestion of more *Anisakis*. A positive relationship between parasitic load in fish and fish size has been identified in previous studies [3,33].

Results obtained from the analysis of the relationship between diet and the total number of *Anisakis* are also difficult to interpret. Although most of the prey species included in the analysis had high infection rates (Appendix A, Table A1), only two of them had a significant effect on the parasitic load. Furthermore, for those that had a significant effect, a decrease in parasitic load was observed as the number of individuals of that species in the stomach contents increased. There are several possible explanations for such counterintuitive relationships, not least that a higher abundance of one prey species in the diet could be associated with a reduction in consumption of another prey species with a higher parasite load or that reduced food intake could be a consequence of adverse effects of high parasite load on individual health. It is notable that fish species with high infection rates such as *M. merluccius* and *T. trachurus* were not significant predictors of parasite load in dolphins. This may be because their importance in the diet is not sufficiently high to have a detectable effect on the overall parasite burden. It is important to note that not all prey species were found in all the stomachs analysed (Table 4 and Table 5). A high number of zeros in the data (many stomachs containing zero individuals of certain prey) would reduce the statistical power of the model to detect an effect of the number eaten. In addition, the effect of eating larger fish on parasite load depends on the shape of the relationship between the size of a fish and its parasite load. Theoretically, eating a higher number of small fish could result in fewer parasites being ingested than if fewer larger fish were eaten. Finally, the relatively small sample size limits our ability to tease out the roles of all the factors affecting parasite load.

It is important to consider the limitations of using stomach content analysis to infer diet, especially at the individual level. The stomach contents of a dead animal are a snapshot of the diet and not necessarily indicative of individual dietary preferences over a longer time scale. Such samples can also be biassed due to differences in the rates of retention, digestion, and degradation of hard remains of different prey species. Furthermore, the sample set involves only dead individuals and represents a very small proportion of the population, meaning it may not accurately reflect the typical feeding habits of healthy dolphins [17,60,61]. It is possible that the prey species that contributed most to infecting the dolphin in question may not be present in the stomach at the time of its death.

Finally, the abundance of *Anisakis* in its final hosts could be considered a reflection of its abundance in the ecosystem as a whole and, as such, the role of physical oceanographic parameters needs to be considered. For example, Mattiucci et al. [3] observed that the survival of *Anisakis simplex* larvae slightly increased with increasing salinity and decreased with increasing temperature, so these two factors may influence the distribution and abundance of this species.

## 5. Conclusions

The abundance of *Anisakis* parasite has generally increased over the years in Galician waters, which has been observed in both cetacean and fish species. However, this trend has been less clear since 2015, which could reflect changes in feeding habits or perhaps the increasing elimination of fishery discards as a source of infection. Parasite loads also appear to undergo seasonal variations, with higher abundance during the spring months. In addition, dolphins dying due to bycatch exhibited a significantly lower parasitic load than those which died of other causes, possibly implying a negative relationship between dolphin health and parasite load.

Diet analysis confirmed that *D. delphis* are primarily piscivorous, with *M. poutassou* being the most important prey species. Other important fish prey species included *M. merluccius, Trisopterus* spp., and the family Gobiidae. Cephalopods were present in the diet but in lower proportions, with species of the genus *Loligo* being the most important.

A positive relationship was observed between parasitic load and cetacean size, most likely because bigger dolphin eat more food. The relatively low number of *Anisakis* seen in most stomachs suggests they do not normally accumulate, and many ingested larvae probably never attach to the stomach wall and are rapidly egested. Conversely, the very high loads seen in a few individuals probably do indicate accumulation and perhaps associated pathology.

Relationships were expected between diet and parasitic load but the only significant relationships seen linked higher consumption of *M. poutassou* (and to a lesser extent *S. scombrus*) to lower parasite load. However, much larger sample sizes may be needed to reliable quantify such relationships.

It is important to consider the limitations of this study, including its reliance on stomach content analysis, which provides only a snapshot of the diet of stranded dolphins, and possible biases associated with differing digestion and retention rates in different prey species may affect the interpretation of dietary composition. Incorporating complementary methods such as stable isotope analysis or metabarcoding could provide a more comprehensive view of the dolphin diet. Obtaining more detailed information on the health status of the analysed dolphins would permit a better understanding of the impact of parasitic infection on their well-being and physiological condition.

## Figures and Tables

**Table 1:** Summary of the *D. delphis* samples analysed in this study.

Year	N	Quarter of the Year				Sex		Evidence of Bycatch	
		1	2	3	4	Female	Male	Yes	No
2004	5			1	4	1	4	2	3
2005	8	6			2	2	6	4	4
2006	4	2	1		1	1	3	3	1
2007	12	4	1	7		3	9	9	3
2008	19	7	10	2		9	10	8	11
2009	6	5	1			3	3		6
2010	0								
2011	1			1			1		1
2012	0								
2013	2	1	1			2		1	1
2014	1	1					1		1
2015	0								
2016	1	1					1		1
2017	7	3	1	2	1	5	2	2	5
2018	12	9		3		4	8	4	8
2019	2			2		2		2	
2020	11	4	1		6	7	4	7	4
2021	10	7		1	2	7	3	4	6
2022	2	2					2	NA	NA
2023	0								
2024	14	14				7	7	NA	NA
TOTAL	117	66	16	19	16	53	64	46	55

**Table 2:** Summary of the estimated numbers and sizes of “adult” (>3.5 cm length) and “larvae” (≤3.5 cm) *Anisakis* present in the subsample of 47 dolphin stomach contents analysed.

Category	Number	% of Total	Median Number	Average Size (cm)	Size Range (cm)
“Adults”	12726	18.5	271	4.51	3.6–8.7
“Larvae”	55935	81.5	1190	2.13	0.3–3.5
TOTAL	68661	100.00	1461	2.98	0.3–8.7

**Table 3:** Detailed diet composition based on the stomach contents of 18 *D. delphis* stranded in Galicia between 2019 and 2024. All prey species are presented in alphabetical order of family names, with fish and cephalopods listed separately. The tables show each dietary index as a number and as a percentage: the frequency of occurrence (F and F%), numerical importance (N and N%), the importance by weight (W and W%) and the Index of Relative Importance (IRI and IRI%), as well as the range and mean for prey length (cm) and estimated prey weight (g).

Prey	F	F%	N	N%	W	W%	IRI	IRI%	Length Range (mm)	Mean Length (mm)	Weight Range (g)	Mean Weight (g)
Fishes												
Ammodytidae												
*Ammodytes* sp.	1	5.56	5	0.57	32.07	0.04	3.36	0.06	116.08–146.74	129.36	4.57–9.03	6.41
*Gymnammodytes* sp.	1	5.56	2	0.23	0.00	0.00	1.27	0.02	NA	NA	NA	NA
Unidentified Ammodytidae	1	5.56	1	0.11	0.00	0.00	0.63	0.01	147.06	147.06	NA	NA
Argentinidae												
*Argentina* sp.	3	16.67	149	16.97	128.60	0.14	285.23	5.37	42.49–62.51	56.23	0.28–1.29	0.86
Atherinidae												
*Atherina* sp.	2	11.11	5	0.57	31.07	0.03	6.71	0.13	99.20–110.55	106.20	6.30–8.72	7.77
Belonidae												
*Belone belone*	2	11.11	3	0.34	0.00	0.00	3.80	0.07	NA	NA	NA	NA
Bothidae												
*Arnoglosus laterna*	1	5.56	3	0.34	3.21	0.00	1.92	0.04	58.03–60.34	58.80	1.02–1.16	1.07
Carangidae												
*Trachurus trachurus*	4	22.22	9	1.03	661.28	0.74	39.16	0.74	49.85–255.99	171.67	0.68–148.10	73.48
Clupeidae												
*Sardina pilchardus*	2	11.11	2	0.23	213.68	0.24	5.18	0.05	226.55–238.81	232.68	98.07–115.60	106.84
Unidentified Clupeidae	1	5.56	1	0.11	16.69	0.02	0.74	0.01	128.44	128.44	16.62	16.62
Gadidae												
*Gadiculus argentus*	2	11.11	33	3.76	6.41	0.01	41.84	0.79	21.03–25.29	22.46	0.001–1.40	0.19
*Pollachius pollachius/P. virens/* *Melanogrammus aeglefinus*	2	11.11	2	0.23	138.54	0.15	4.25	0.09	79.74–233.22	156.48	6.26–132.28	69.27
*Micromesistius poutassou*	6	33.33	238	27.11	82,395.41	91.87	3965.77	74.84	56.79–293.98	177.11	6.44–1571.49	346.20
*Trisopterus luscus*	2	11.11	26	2.96	660.09	0.74	41.08	0.77	3.58–301.03	46.27	0.0004–362.28	25.39
*Trisopterus luscus/T. minutus*	1	5.56	1	0.11	4.55	0.01	0.66	0.01	71.60	71.60	4.55	4.55
*Trisopterus* sp.	3	16.67	80	9.11	282.05	0.31	157.10	2.96	43.34–146.19	79.23	0.41–25.82	3.53
Total *Trisopterus*	4	22.22	107	12.19	946.68	1.06	198.84	3.74	3.58–301.03	71.38	0.0004–362.28	8.93
Unidentified Gadidae	2	11.11	10	1.14	156.26	0.17	14.59	0.27	21.03–25.29	22.46	0.001–1.40	0.19
Gobiidae												
*Gobius* sp.	3	16.67	66	7.52	96.20	0.11	127.07	2.39	35.31–85.43	57.10	0.23–6.29	1.46
*Pomatoschistus* sp.	1	5.56	5	0.57	1.80	0.00	3.17	0.06	1.95–54.52	35.47	0.0006–0.76	0.36
Unidentified Gobiidae	2	11.11	81	9.23	46.92	0.05	103.09	1.94	29.04–52.02	45.20	0.12–0.95	0.58
Merlucciidae												
*Merluccius merluccius*	5	27.78	25	2.85	2117.04	2.36	144.66	2.73	139.86–319.42	218.15	21.18–234.83	84.68
Scombriidae												
*Scomber scombrus*	1	5.56	1	0.11	11.75	0.01	0.71	0.01	137.25	137.25	11.75	11.75
Soleidae												
*Solea solea*	1	5.56	1	0.11	124.80	0.14	1.41	0.03	238.17	238.17	124.80	124.80
Sparidae												
*Boops boops*	1	5.56	7	0.80	412.98	0.46	6.99	0.13	161.95–212.82	187.25	37.99–83.69	59.00
Unidentified Sparidae	1	5.56	1	0.11	0.00	0.00	0.63	0.01	NA	NA	NA	NA
Sternoptychidae												
*Maurolicus muelleri*	3	16.67	4	0.46	9.92	0.01	7.78	0.14	57.90–64.44	60.73	2.11–2.98	2.48
Trachinidae												
*Echiichthys vipera*	1	5.56	1	0.11	4.36	0.00	0.66	0.01	76.34	76.34	4.36	4.36
Unidentified flat fishes	2	11.11	2	0.23	0.00	0.00	2.53	0.05	NA	NA	NA	NA
Unidentified fishes	3	16.67	7	0.80	0.00	0.00	13.29	0.25	NA	NA	NA	NA
Cephalopods												
Eledonidae												
*Eledone moschata*	1	5.56	1	0.11	81.71	0.09	1.14	0.02	103.77	103.77	81.71	81.71
*Eledone* sp.	1	5.56	3	0.34	1.88	0.00	1.91	0.04	4.61–9.12	7.62	0.39–0.74	0.63
Gonatidae												
*Gonatus* sp.	1	5.56	3	0.34	526.89	0.59	5.16	0.10	192.39–218.11	202.39	151.69–214.13	175.63
Histioteuthidae												
*Histioteuthis* sp.	1	5.56	1	0.11	86.69	0.10	1.17	0.02	70.80	70.80	86.69	86.69
Loliginidae												
*Alloteuthis* sp.	5	27.78	10	1.14	22.92	0.03	32.35	0.61	20.19–82.98	57.89	2.65–7.39	4.58
*Loligo* sp.	6	33.33	47	5.35	1239.48	1.38	224.50	4.23	4.13–278.02	56.98	0.31–469.60	49.28
Unidentified Loliginidae	1	5.56	1	0.11	5.25	0.01	0.67	0.01	37.84	37.84	5.25	5.25
Ommastrephidae	3	16.67	15	1.71	140.73	0.16	31.09	0.59	21.79–96.24	49.91	1.16–32.69	9.38
Sepiolidae												
*Sepietta* sp.	2	11.11	3	0.34	7.04	0.01	3.88	0.07	23.59–23.97	23.78	3.23–3.81	3.52
*Sepiola* sp.	1	5.56	9	1.03	13.31	0.01	5.78	0.11	19.37–20.03	19.53	1.43–1.61	1.48
Unidentified Sepiolidae	2	11.11	4	0.46	9.11	0.01	5.17	0.10	19.61–20.19	19.80	2.10–2.65	2.28
Unidentified cephalopods	1	5.56	9	1.03	0.00	0.00	5.69	0.11	NA	NA	NA	NA
Crustaceans												
Isopoda												
TOTAL	18		878	100	89,464.05	100	5309.13	100				

**Table 4:** Dietary indices of the main prey species in the stomach contents of 90 *D. delphis* stranded in Galicia between 2004 and 2024. The tables show each dietary index as a number and as a percentage: the frequency of occurrence (F and F%), numerical importance (N and N%), the importance by weight (W and W%) and the Index of Relative Importance (IRI and IRI%).

Prey	F	F%	N	N%	W	W%	IRI	%IRI
Clupeidae	32	35.56	233	5.38	975.00	0.01	191.56	2.35
*Merluccius merluccius*	40	44.44	256	5.91	1,730,397.31	9.38	679.64	8.35
*Micromesistius poutassou*	39	43.33	3190	73.69	13,950,990.31	75.61	6469.65	79.46
*Scombrus scombrus*	20	22.22	64	1.48	2,221,997.75	12.04	300.47	3.69
*Trachurus trachurus*	31	34.44	211	4.87	273,424.28	1.48	218.93	2.69
*Trisopterus* spp.	25	27.78	375	8.66	273,326.68	1.48	281.77	3.46
TOTAL	90		4329	100	18,451,111.34	100	8142.02	100

**Table 5:** Optimal models for (GAM 1) baseline variables, (GAM 2) baseline variables with the bycatch variable, and (GAM 3) baseline variables with diet variables. The models are shown with the significant explanatory variables, their Akaike value (AIC), the deviance explained for each model, and the sample size (N).

No.	Model	AIC	Explained Deviance	N
GAM 1	Total *Anisakis* ~ s(year, k = 4) + s(month, bs = “cc”) + s(length, k = 4)	1704.176	29.9%	117
GAM 2	Total *Anisakis* ~ s(year, k = 4) + s(month, bs = “cc”) + s(length, k = 4) + as.factor(bycatch)	1428.308	44.5%	101
GAM 3	Total *Anisakis* ~ s(year, k = 4) + s(length, k = 4) + s(N_MAC, k = 4) + s(N_WHB, k = 4)	903.905	37.3%	90

## Data Availability

The datasets presented in this article are part of an ongoing study. Requests to access the data should be directed to erueda@iim.csic.es.

## Abbreviations

The following abbreviations are used in this manuscript:

AIC	Akaike information criterion
CEMMA	Coordinadora para o Estudo dos Mamíferos Mariños
CLP	Clupeidae
COD	Cause of death
GAM	Generalised additive model
HKE	*Merluccius merluccius* (hake)
HOM	*Trachurus trachurus* (horse mackerel)
MAC	*Scomber scombrus* (mackerel)
NGO	Non-governmental organisation
TRX	*Trisopterus* spp. (poor cod)
WHB	*Micromesistius poutassou* (blue whiting)

## Appendix A

**Table A1:** *Anisakis* parasitisation in main prey species of *D. delphis*. The table includes Zone (sample zone); Tissue (fish tissue sampled); Parasitological indices: P% = prevalence, A = mean abundance, and I = mean intensity (of infection, i.e., the mean of non-zero values); Year of publication; and Source. Abbreviations in Zone indicate the following: NA = North Atlantic; NEA = Northeast Atlantic; AIB = Atlantic Iberian Peninsula; NWIB = Northwest Iberian Peninsula; NS = North Sea; Med = Mediterranean. Abbreviations in Tissue indicate the following: M = musculature; V = viscera. Other abbreviations: NG = North Galicia; SG = South Galicia; Y00 = 2000; Y01 = 2001. Numbers appearing in parentheses are 95% confidence intervals.

Species	Zone	Tissue	P%	A	I	Year	Source
*Sardina pilchardus*	Galicia	Total	10	-	1	1994	[62]
	Galicia	Total	0	0	0	2001	[21]
	Galicia	Total	28.3	-	1.82	2015	[63]
	Galicia	Total	62.14	1.91	3.07	2018	[26]
	NA	Total	11.43	0.34 (±0.26)	-	2019	[24]
	NEA	Total	9.71	0.29 (±1.44)	2.94 (±3.77)	2022	[64]
*Trachurus trachurus*	Galicia	Total	88	13.57 (±21.73)	15.51 (±22.58)	2001	[21]
	Galica	Total	88 (Y00) 96 (Y01)	-	8 18.3	2008	[22]
	Portugal	Total	67.31	14.46	21.48	2014	[23]
	NA	Total	100	52.50 (±17.24)	-	2019	[24]
*Scomber scombrus*	Galicia	Total	74.54	4.61	6.15	2001	[21]
	AIB	Total	87.3	7.7	8.8	2018	[25]
	Galicia	Total	86.31	7.36	8.52	2018	[26]
	NA	Total	76.43	7.29 (±3.13)	-	2019	[24]
*Merluccius merluccius*	Galicia	Total	100	11.4	14.25	2001	[21]
	Galicia	Total	98.5	30	-	2015	[27]
	Galicia	M V	95.9 96.0	60.1 66.3	62.6 69.1	2018	[28]
	Galicia	Total	97.98	126.42	129.03	2018	[26]
	NA	Total	84.00	20.34 (±19.94)	-	2019	[24]
	NEA	Total	88	51.3	-	2022	[29]
	Portugal	Total	95.6	10.5	11	2022	[30]
*Micromesistius poutassou*	Galicia	Total	91.16	4.61	5.06	2001	[21]
	Galicia	Total	99.3	-	11.1	2005	[31]
	AIB	M V	50.5 96.9	2.1 (±5.6) 2.41 (±70)	4.1 (±7.3) 24.9 (±71.9)	2018	[33]
	Galicia	Total	99.25	34.89	35.15	2018	[26]
	NA	Total	77.51	4.83 (±1.75)	-	2019	[24]
	NWIB	Total	84.1	31.3	39.3	2020	[32]
*Trisopterus luscus*	Galicia	Total	32	1	2.67	2001	[21]
	Portugal	V	20.7	-	0.21	2003	[65]
Loliginidae	Galicia	Total	0	-	-	2001	[21]
*Loligo forbesii*	NS		8.8	0.1	1.1	2026	[66]
Ommastrephidae	Galicia	Total	15.8	-	-	2001	[21]
*Illex coindetii*	Galicia		19.3 (NG) 2.7 (SG)	2.8 <0.1	5.9 1.5	1995	[67]
	Galicia	Total	11.7	1.01	5.56	2001	[21]
	Med		12.2	0.2	1.8	2016	[68]
	NS		28.3	0.5	1.9	2026	[66]

## Appendix B

**Table A2:** Regression equations for calculating the length (L) and weight (W) of prey. The following measurements have been used: DL = dorsal line of the dentary, LHL = hood length (cephalopods), LRL = rostrum length (cephalopods), OF = external fork, OL = otolith length, OW = otolith width, PMXHH = premaxilla height, PMXL = premaxilla length, S = dentary symphysis height, VERL = vertebra length, and VL = ventral line of the dentary. The sources used are as follows: Be = Bedford et al. (1986) [69], Bo = Borges et al. (2003) [70], BP1 = Brown and Pierce (1997) [71], BP2 = Brown and Pierce (1998) [40], CL = Clarke (1986) [41], Co = Coull et al. (1989) [72], GH = Grellier and Hammond (2006) [48], Ha = Harkönen (1986) [36], He1 = Hernández-Milian (2005) [73], He2 = Hernández-Milian (2015) [74], He(up) = Hernández-Milián unpublished, HG = Hernández-González (2023) [18], MN = Marçalo and Nicolau (2018) [20], O’L = O’Leary (2009), Pe = Pedà et al. (2022) [43], Sa1 = Santos et al. (2007) [75], and Wa = Watt et al. (1997) [38].

Species	Estimated Size (mm)	Source	Estimated Weight (g)	Source
FISHES				
Ammodytidae	L = VERL/0.017	Wa		
*Ammodytes* spp.	L = 8.776 + 51.096 × OL	Ha	W = 0.61215 × OL^2.71^	Ha
*Argentina* spp.	L = 10.466 + 40.03 × OL	Ha	W = 0.5592 × OL^3.173^	Ha
*Arnoglossus* spp.	L = −2.038 + 46.205 × OL	HG	W = 0.4223713 × OL^3.3685^	HG
*Atherina* spp.	L = 67.42 + 15.132 × OL	BP2	W = 0.006304 × (L/10)^3.01^	BP2
*Boops boops*	L = 71.789 + OL × 18.081	MN	W = 0.01208 × (L/10)^2.892^	Bo
*Echiichthys vipera*	L = 25.484 × OL − 17.955	AFORO	W = 0.0098 × (L/10)^3.00^	Fishbase
*Gadiculus argentus*	L = 19.449 + 1.053 × OL	Ha	W = 0.0021289 × OL^3.785^	Ha
Gadidae (unident.)	L = −61.59 + 33.304 × OL	BP2	W = 0.016042 × (L/10)^2.87419^	BP2
	L = 80.79 + 81.681 × S	He2		
Gobiidae	L = −6.46 + 41.77 × OL	Ha	W = 0.232809 × OL^4.17^	Ha
*Gobius*	L = −6.46 + 41.77 × OL	Ha	W = 0.232809 × OL^4.18^	Ha
*Maurolicus muelleri*	L = 25.207 + 26.152 × OW	O’L	W = 0.377 × OW^2.05^	O’L
	L = (VL × 60)/4.39	He (up)	W = 0.0074 × (L/10)^3.22^	Fishbase
*Merluccius merluccius*	L = −0.63 + 23.884 × OL	Ha	W = 0.00974 × (L/10)^2.913^	Be
	lnL = 4.5918 + 0.9314 × lnPMXHH	Wa		
	L = 32 + 12.4 × OF	He1		
*Micromesistius poutassou*	L = −40.94 + 25.394 × OL	Ha	W = 0.01935 × (L/10)^3.34372^	Co
	L = −17.800 + 70.77 × OW	Ha		
	L = 30.71 + 13.21 × PMXL	Wa		
	L = 80.79 + 81.681 × S	He2		
*Pollachius pollachius/* *P. virens/Melanogrammus aeglefinus*	L = −116.49 + 38.857 × OL	BP1	W = 0.01798 × (L/10)^2.827^	BP1
*Pomatoschistus* spp.	L = −3.89 + 44.93 × OW	Ha	W = 0.3345 × OW^3.121^	Ha
*Sardina pilchardus*	L = −23.972 + 12.907 × VL	He (up)	W = 0.0058 × (L/10)^3.12^	Fishbase
*Scomber scombrus*	L = −20.41 + 87.59 × OL	Ha	W = 1.094 × OL^4.039^	Ha
*Solea solea*	L = −12.622 + 80.901 × OL	Ha	W = 2.535 × OL^3.444^	Ha
*Trachurus trachurus*	L = −27.02 + 34.939 × OL	BP	W = 0.003400 × (L/10)^3.29430^	Co
	L = 13.213 × DL − 1.1402	He2		
	L = 13.65 × VERL − 23.83	He2		
*Trisopterus luscus*	L = −160.42 + 41.95 × OL	Ha	W = 0.0105 × (L/10)^3.069^	Be
	L = −39.6 + 61.683 × OW	Ha		
*Trisopterus luscus/T. minutus*	L = −109.1 + 36.139 × OL	Ha	W = 0.00079 × OL^5.38^	Ha
*Trisopterus* spp.	L = −5.886 + 23.443 × OL	BP	W = 0.002796 × (L/10)^3.404^	Ha + Sa1
	L = 0.6593 + 109.4232 × S	He2		
CEPHALOPODS				
*Alloteuthis* spp.	L = −30.99 + 113.97 × LRL	Cl	lnW= 2 + 2.75 × lnLRL	Cl
*Eledone moschata*	L = −13.418 + 45.072 × LHL	Pe	lnW= 1.68 + 2.85 × lnLHL	Cl
*Eledone* spp.			lnW= 1.68 + 2.85 × lnLHL	Cl
*Gonatus* spp.	L = −43.4 + 42.87 × LRL	Cl	lnW = −0.655 + 3.33 × lnLRL	Cl
*Histiuteuthis* spp.	L = −13.6 + 22.21 × LRL	Cl	lnW = 1.779 + 2.01 × lnLRL	Cl
*Loligo* spp.	L = −42.22 + 84.274 × LRL	Cl	W = 6.19536 × LRL^3.242^	Cl
Ommastrephidae	L = −11.3 + 41.36 × LRL	Cl	W = 2.18803 × (LRL)^2.83^	Cl
*Sepietta* spp.	L = 22.06 + 2.55 × LHL	Cl	lnW = 1.55 + 0.74 × lnLHL	Cl
*Sepiola* spp.	L = 18.54 + 1.65 × LRL	Cl	W = 0.64545 + LRL^0.35^	Cl
Sepiolidae	L = 18.54 + 1.65 × LHL	Cl	W = 2.65117 × LHL^0.545^	Cl

## Appendix C

**Table A3:** Diet composition of the stomach contents of 18 *D. delphis* stranded in Galicia between 2019 and 2024 by (**a**) class (fishes, cephalopods and crustaceans) and (**b**) family. F is the frequency of occurrence and F% its percentage, N is the numerical prey importance and N% its percentage, W is the reconstructed weight prey importance and W% its percentage, and IRI is the Index of Relative Importance and IRI% its percentage; reconstructed length range and mean is given in centimetres (cm) and reconstructed weight range and mean in grams (g).

(a)												
Prey	F	F%	N	N%	W	W%	IRI	IRI%	Length Range (cm)	Mean Length (cm)	Weight Range (g)	Mean Weight (g)
Fishes	16	88.89	771.00	87.81	87,334.29	97.62	16,482.90	96.25	1.95–319.42	103.46	0.0004–1571.49	116.45
Cephalopods	8	44.44	106.00	12.07	2129.76	2.38	642.38	3.75	4.13–278.02	54.56	0.31–469.60	32.62
Crustaceans	1	5.56	1	0.11	0	0.00	0.63	0.00	NA	NA	NA	NA
TOTAL	18		878.00	100.00	89,464.05	100.00	17,125.91	100.00				
**(b)**												
Fishes												
Ammodytidae	3	16.67	8	0.91	32.07	0.04	15.78	0.16	116.08–147.06	132.31	4.57–9.03	6.41
Argentinidae	3	16.67	149	16.97	128.60	0.14	285.24	2.93	42.49–62.51	56.23	0.28–1.29	0.86
Atherinidae	2	11.11	5	0.57	31.07	0.03	6.71	0.07	99.20–110.55	106.20	6.30–8.72	7.77
Belonidae	2	11.11	3	0.34	0.00	0.00	3.80	0.04	NA	NA	NA	NA
Bothidae	1	5.56	3	0.34	3.21	0.00	1.92	0.02	58.03–60.34	58.80	1.02–1.16	1.07
Carangidae	4	22.22	9	1.03	661.28	0.74	39.20	0.40	49.85–255.99	171.67	0.68–148.10	73.48
Clupeidae	3	16.67	3	0.34	16.69	0.02	6.01	0.06	128.44–238.81	197.93	16.62–115.60	76.76
Gadidae	11	61.11	390	44.42	83,643.30	93.49	8428.01	86.71	2.94–301.03	131.74	0.0004–1571.49	215.02
Gobiidae	4	22.22	152	17.31	144.92	0.16	388.31	4.00	1.95–85.43	50.05	0.0006–6.29	0.95
Merlucciidae	5	27.78	25	2.85	2117.04	2.37	144.83	1.49	139.86–319.42	218.15	21.18–234.83	84.68
Scombriidae	1	5.56	1	0.11	11.75	0.01	0.71	0.01	137.25	137.25	11.75	11.75
Soleidae	1	5.56	1	0.11	124.80	0.14	1.41	0.01	238.17	238.17	124.80	124.80
Sparidae	2	11.11	8	0.91	412.98	0.46	15.25	0.16	161.95–212.82	187.25	37.99–83.69	59.00
Sternoptychidae	3	16.67	4	0.46	2.21	0.00	7.63	0.08	57.90–64.44	60.73	2.11–2.98	2.48
Trachinidae	1	5.56	1	0.11	4.36	0.00	0.66	0.01	76.34	76.34	4.36	4.36
Flat fish	2	11.11	2	0.23	0.00	0.00	2.53	0.03	NA	NA	NA	NA
Unidentified fish	3	16.67	7	0.80	0.00	0.00	13.29	0.14	NA	NA	NA	NA
Cephalopods												
Eledonidae	2	11.11	4	0.46	83.59	0.09	6.10	0.06	4.61–103.77	39.17	0.39–81.71	27.61
Gonatidae	1	5.56	3	0.34	526.89	0.59	5.17	0.05	192.39–218.11	202.39	151.69–214.13	175.63
Histioteuthidae	1	5.56	1	0.11	86.69	0.10	1.17	0.01	70.80	70.80	86.69	86.69
Loliginidae	6	33.33	58	6.61	1262.40	1.41	267.23	2.75	4.13–278.02	57.13	0.31–469.60	41.83
Ommastrephidae	3	16.67	15	1.71	140.73	0.16	31.10	0.32	21.79–96.24	49.91	1.16–32.69	9.38
Sepiolidae	4	22.22	16	1.82	29.46	0.03	41.23	0.42	19.37–23.97	20.17	1.43–3.81	1.96
Unidentified Cephalopods	1	5.56	9	1.03	0.00	0.00	5.69	0.06	NA	NA	NA	NA
Crustaceans												
Isopoda	1	5.56	1	0.11	0.00	0.00	0.63	0.01	NA	NA	NA	NA
TOTAL	18		878	100	89,464.05	100	9719.61	100				
